# Optimization of black-box models with uncertain climatic inputs—Application to sunflower ideotype design

**DOI:** 10.1371/journal.pone.0176815

**Published:** 2017-05-25

**Authors:** Victor Picheny, Ronan Trépos, Pierre Casadebaig

**Affiliations:** 1 MIAT, Université de Toulouse, INRA, Castanet-Tolosan, France; 2 AGIR, Université de Toulouse, INRA, Castanet-Tolosan, France; Nankai University, CHINA

## Abstract

Accounting for the interannual climatic variations is a well-known issue for simulation-based studies of environmental systems. It often requires intensive sampling (e.g., averaging the simulation outputs over many climatic series), which hinders many sequential processes, in particular optimization algorithms. We propose here an approach based on a subset selection in a large basis of climatic series, using an ad-hoc similarity function and clustering. A non-parametric reconstruction technique is introduced to estimate accurately the distribution of the output of interest using only the subset sampling. The proposed strategy is non-intrusive and generic (i.e. transposable to most models with climatic data inputs), and can be combined to most “off-the-shelf” optimization solvers. We apply our approach to sunflower ideotype design using the crop model SUNFLO. The underlying optimization problem is formulated as a multi-objective one to account for risk-aversion. Our approach achieves good performances even for limited computational budgets, outperforming significantly standard strategies.

## 1 Introduction

Using numerical models of complex dynamic systems has become a central process in many fields, including engineering or natural sciences. It is now an essential tool for water resource management, adaptation of anthropic or natural systems to a changing climatic context or the conception of new production systems.

Many times, the objective pursued by model users amounts to solving an optimization problem, that is, find the set of input parameters of the model that maximizes (or minimizes) the output of interest (cost, production level, environmental impact, etc.). Examples of such problems abound with environmental models, including water distribution systems design [1] or agricultural watershed management [2]. In agronomy, in the past two decades crop models have received a growing attention [3–6], as they can be used to help improve the plant performances, either through cultural practices [7, 8] or model-assisted plant breeding [9, 10].

Within the wide range of potential approaches to solve such optimization problems, *black-box optimization methods* have proven to be popular in this context [9, 11], in particular because they are in essence non-intrusive: they only require pointwise evaluations of the model at hand (output value for a given set of inputs), as opposed to knowing the underlying mechanisms of the model, derivative information, etc. This greatly facilitates implementation and avoids developping taylored algorithms.

However, a well-known difficulty, shared by many agricultural or ecological models users, lies in dealing with climatic information. Many models require series of measures of precipitation, temperature, etc., as input variables: typically, a crop model requires day-to-day measures over the agricultural season. Those inputs are particularly crucial for agricultural or ecological models, for which the climate has a preponderant impact on the system. To avoid drawing conclusions biased by the choice of a particular set (e.g., year) of climatic data, scenarios approaches can be used, duplicating the analysis for a small number of distinct climates [12, 13]. Alternatively, one may compute utility functions based on the model outputs over a (large) number of climatic datasets [14, 15]. This avoids the potentially challenging task of identifying scenarios, but requires intensive computation, as the number of datasets must be large enough to obtain a stable estimation of the utility (see for instance [8] for a discussion on uncertainty propagation on agro-ecosystem models). This approach rapidly becomes computationally prohibitive if the analysis is embedded in an optimization loop, even for moderately complex models.

A natural solution is to treat the climate as a random variable, which allows the use of the robust (or noisy) optimization framework (see e.g. [1]). However, if readily available codes abound for continuous, box-constrained parameters and deterministic outputs, solutions become scarce for systems depending on stochastic phenomena. Besides, the problem formulation becomes more complex, as typically risk-aversion preferences need to be accounted for [8, 15].

In this work, we propose to address the issue of propagating climatic uncertainties in an optimization algorithm with a reasonable computational cost. Our approach is based on a subset selection in a large basis of climatic series (Section 3.1). A non-parametric reconstruction technique is introduced to estimate accurately the distribution of the output of interest based on this subset (Section 3.2). Our solution is designed as non-intrusive and generic, i.e. transposable to most models with climatic data inputs and to most black-box optimization solvers, while allowing parallel computing.

As an application problem, which we use as a running example through this article, we consider the optimization of phenotypes of sunflower (or *ideotype design*, see [11] for a review of recent developments in this research area). Plant performance (e.g., yield), computed using the crop model SUNFLO [16], is maximized with respect to its morphological and/or physiological traits. To account for risk-aversion, the problem is formulated as a multi-objective one.

The rest of this paper is organized as follow: Section 2 briefly reviews previous work on phenotype optimization, describes the SUNFLO model and the multi-objective optimization formulation to solve the problem at hand. Section 3 is dedicated to the optimization algorithm. Sections 4 provides the experimental setup and 5 numerical results.

## 2 Sunflower phenotype optimization

In this section, we first describe briefly the SUNFLO model and corresponding climatic data. Then, we define an optimization problem to account for climatic uncertainty.

### 2.1 Model definition

SUNFLO is a process-based model which was developed to simulate sunflower grain yield (in tons per hectare) and oil concentration as a function of climatic time series, environment (soil and climate), management practices and genetic diversity [16]. It is implemented in the RECORD project [5] which is dedicated to agro-ecosystems studies. It allows to assess the performance of sunflower cultivars in agronomic conditions, hence can be used to perform varietal selection or design.

A sunflower cultivar is represented by a combination of eight genetic coefficients, which are the inputs to be optimized. They describe various aspects of crop structure or functioning: phenology, plant architecture, response curve of physiological processes to drought and biomass allocation. We assume that the coefficients can take continuous values between a lower and an upper bound, determined from a dataset of existing cultivars. The variables and their domain of variation are reported in Table 1.

**Table 1:** Phenotypic coefficients and the bounds used for optimization.

*Description, name and unit*	*Min*	Max
Temperature sum from emergence to the beginning of flowering (TDF1, °C)	765	907
Temperature sum from emergence to seed physiological maturity (TDM3, °C)	1540	1830
Number of leaves at flowering (TLN)	22.2	36.7
Light extinction coefficient during vegetative growth (K)	0.780	0.950
Rank of the largest leave of leaf profile at flowering (LLH)	13.5	20.6
Area of the largest leave of leaf profile at flowering (LLS, *cm*^2^)	334	670
Threshold for leaf expansion response to water stress (LE)	-15.6	-2.31
Threshold for stomatal conductance response to water stress (TR)	-14.2	-5.81

**Fig 1:**
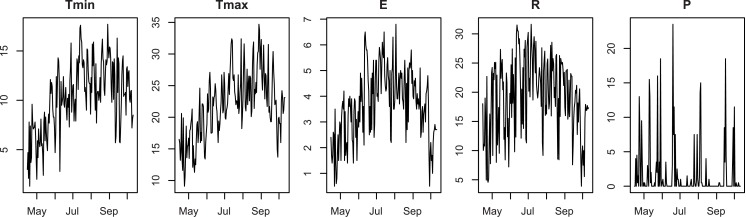
Dataset of the year 2009, Lusignan.

As climatic inputs, SUNFLO uses daily measures over a year of five variables: minimal and maximal temperatures (*T*_min_ and *T*_max_, °C), global incident radiation (*R*, *MJ*/*m*^2^), evapotranspiration (*E*, mm, Penman-Monteith [17]) and precipitations (*P*, mm). Note that the model actually uses only the data corresponding to the cultural year (for sunflower, April to October, 180 days). Fig 1 provides an example of such data.

We denote $x\in X\subset R^{d}$ a particular phenotype, X being the hyper-rectangle defined by the bounds, and *c* = {*T*_min_, *T*_max_, *R*, *E*, *P*} ∈ Ω a given climatic series. Here, *c* can be seen as a matrix of size 5 × 180. Hence, the yield can be seen as a function of the phenotype and the climatic series:

$$\begin{array}{ccc} y:X\times \Omega & \to & R^{+}\\ x,c & ⟼ & y(x,c). \end{array}$$ (1)

In Section 3, we denote by *y*(**X**, **C**) ≔ (*y*(**x**_*i*_, *c*_*j*_))_1 ≤ *i* ≤ *I*, 1 ≤ *j* ≤ *J*_ the *I* × *J* matrix of yield values for a set of phenotypes **X** = {**x**_1_, …,**x**_*I*_} and a set of climatic series **C** = {*c*_1_, …, *c*_*J*_}.

In the following, we consider that the set of climatic series Ω is discrete, since we use historic climatic data (as opposed to using a stochastic generator for instance [18]). We note: *Card*(Ω) = *N* the number of available series for the study.

### 2.2 Optimization under climatic uncertainty

From a farmer point of view, the objective would be to find a phenotype that maximizes the yield for the year to come, without knowing in advance the climate data. Let *C* be the climatic series of the upcoming year (the upper case denoting a random variable); we consider in the following that *C* is uniformly distributed over Ω. The yield *y*(**x**, *C*) is then also a random variable (which we denote in the following *Y*(**x**)), which makes its direct maximization with respect to **x** meaningless. A natural formulation is to maximize the yield expectation:

$$\left(P_{E}\right)\;\max_{x\in X}E\left[y(x,C)\right]=\max_{x\in X}E\left[Y(x)\right]=\max_{x\in X}\frac{1}{N}\sum_{i=1}^{N}y\left(x,c_{i}\right).$$ (2)

However, in general, a farmer also wishes to integrate some prevention against risk in its decision. Such a problem is often referred to as *robust optimization* in the engineering literature (see for instance [19] for a review). In econometrics, this is many times handled by utility functions [20, 21], which offer an automated trade-off between average performance and risk aversion. However, in order to obtain several solutions ranging from risk neutral to highly risk averse, a solution is to consider this problem as multi-objective, by introducing a second criterion to maximize that accounts for the risk [15]. We consider the conditional value-at-risk (CVaR, [22]), defined as:

$${\text{CVaR}}_{\alpha }\left[Y(x)\right]=E\left[Y\left(x\right)|Y\left(x\right)\leq Q_{\alpha }\left[Y(x)\right]\right].$$ (3)

For the sunflower application, CVaR_*α*_ is the average yield over the (*N* × *α*)-th worst years with the usual definition of the quantile: $P[Y\leq Q_{\alpha }\left(Y\right)]=\alpha$, and *α* ∈ (0, 0.5]. Note that in general, a CVaR provides an information that is close to the one of a quantile, but enjoys better stability anbd regularity [22].

The multi-objective optimization problem is then:

$$\left(P_{EC}\right)\;\left\{\begin{array}{cc} \max_{x\in X} & E\left[Y(x)\right]\\ \max_{x\in X} & {\text{CVaR}}_{\alpha }\left[Y(x)\right] \end{array}\right.$$ (4)

## 3 Optimization with a representative subset

The two objective functions, E[Y(x)] and CVaR_*α*_[*Y*(**x**)], require running the SUNFLO simulator *N* times every time a new phenotype **x** is evaluated. Embedded in an optimization loop, which typically requires thousands to millions of calls to the objective functions, this evaluation step becomes prohibitive. We propose to address this problem by replacing the large climatic dataset Ω by a small representative set Ω_*K*_. Ω_*K*_ is chosen prior to optimization (Section 3.1); then, the optimization algorithm is run using Ω_*K*_ and a specific inference strategy (Section 3.2).

### 3.1 Choosing a representative subset of climatic data

To select our subset, we propose to define a distance (or, conversely, a similarity) between two climatic series, then choose a set of series *far from each other* using clustering algorithms.

#### 3.1.1 Computing dissimilarities between climatic time series

**Fig 2:**
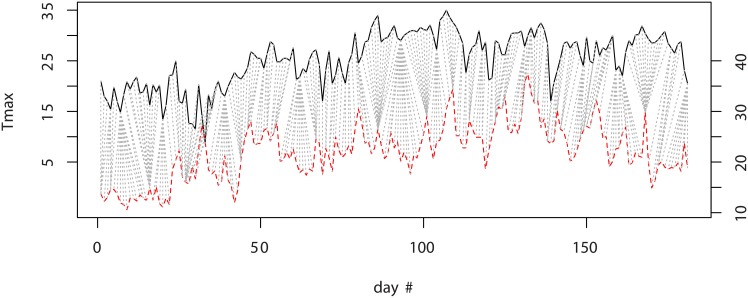
DTW algorithm applied to two series of *Tmax*: Avignon in 1985 (upper curve, left scale) and Lusignan in 2012 (bottom curve, right scale). The dotted lines represent the optimal matching computed by DTW, for a window size of seven days.

A classical tool for time series analysis is an algorithm called Dynamic Time Warping (DTW, [23]). In short, DTW computes distances between time series, allowing two time series that are similar but locally out of phase to align in a non-linear manner, by matching events within a given window. Fig 2 illustrates this concept on two *Tmax* curves. This feature is critical in our context, as for instance two rain time series containing both a large rain event at close dates will have a similar effect on crop, hence should be considered as close to each other.

Given two climatic series *c*_*i*_ and *c*_*j*_, five distances can be computed with DTW, one for each variable: *d*(*c*_*i*_, *c*_*j*_)^*Tmin*^, *d*(*c*_*i*_, *c*_*j*_)^*Tmax*^, *d*(*c*_*i*_, *c*_*j*_)^*R*^, *d*(*c*_*i*_, *c*_*j*_)^*E*^ and *d*(*c*_*i*_, *c*_*j*_)^*P*^.

However, these distances are not sufficient, since two climatic series can be far from each other with respect to the DTW distance yet lead to the same outputs if some key features with respect to the model are similar. Unfortunately, these features are problem-dependent and in general unknown, even to experts. Hence, we propose to define a sixth, model-dependent distance. To do so, we choose first a small set of *l* inputs: $B=\{x_{1},\ldots ,x_{l}\}$. Typically, B can be chosen by Latin Hypercube Sampling (LHS, [24]) to “fill” the search space $R^{d}$ and obtain a large variety of input values. For this set, the output is computed for all the climatic series. Then, the model-based distance is simply the Euclidean distance with respect to *y*:

$$d{\left(c_{i},c_{j}\right)}^{M}=\sqrt{\frac{1}{l}\sum_{k=1}^{l}{\left(y\left(x_{k},c_{i}\right)-y\left(x_{k},c_{j}\right)\right)}^{2}}.$$ (5)

Note that this distance strongly depends on the choice of B. Based on a space-filling design, it aims at being relevant on average over X, but might be misleading locally. We address this specific point in Section 3.3.

To avoid scaling issues and attribute equal importance to all variables, we use the normalization procedure described in [25], which works as follow: Let **D** be a *N* × *N* matrix of distances (with values *d*_*ij*_ = *d*(**x**_*i*_, **x**_*j*_), *d*_*ij*_ = *d*_*jj*_ and *d*_*ii*_ = 0). We first compute a corresponding similarity matrix **S**, with values:

$$s_{ij}=-\frac{1}{2}\left[d_{ij}-\frac{1}{N}\sum_{k=1}^{N}\left(d_{ik}+d_{kj}\right)+\sum_{k=1}^{N}\sum_{k^{\prime }=1}^{N}d_{kk^{\prime }}\right].$$

Then, we normalize **S** with:

$${\bar{s}}_{ij}=\frac{s_{ij}}{\sqrt{s_{ii}+s_{jj}}},$$

and the normalized dissimilarity matrix $\bar{D}$ has elements defined as:

$${\bar{d}}_{ij}={\bar{s}}_{ii}+{\bar{s}}_{jj}-2{\bar{s}}_{ij}=2-2{\bar{s}}_{ij}.$$

Finally, we combine the six dissimilarities into a single scalar using a convex combination:

$$\delta_{ij}=\alpha_{T_{\min}}{\bar{d}}_{ij}^{T_{\min}}+\alpha_{T_{\max}}{\bar{d}}_{ij}^{T_{\max}}+\alpha_{P}{\bar{d}}_{ij}^{P}+\alpha_{E}{\bar{d}}_{ij}^{E}+\alpha_{S}{\bar{d}}_{ij}^{S}+\alpha_{M}{\bar{d}}_{ij}^{M},$$ (6)

with $\alpha_{T_{\min}}+\ldots +\alpha_{M}=1$. In the following, we use $\alpha_{M}=1/2$ and the other weights equal to 1/10, to give an equal weight to the model-based distance and the DWT distances.

#### 3.1.2 Choosing a representative subset using classification

Once the matrix of dissimilarities (*δ*_*ij*_)_1 ≤ *i*, *j* ≤ *N*_ is computed, most unsupervised clustering algorithms can be used to split the set of climatic series Ω into subsets. However, a difficulty here is that the centroids of the clusters cannot be computed. Hence, we use a variation of the k-means algorithm that only requires *dissimilarities* to the centroids. We follow the approach described in [25].

The algorithm divides the set Ω into *K* classes $C^{1},\ldots ,C^{K}$. A class $C^{k}$ contains *N*^*k*^ elements $\left\{c_{1}^{k},\ldots ,c_{K}^{k}\right\}$. Any element *c* ∈ Ω is uniquely attributed to one class and we have: $\sum_{k=1}^{K}N^{k}=N$. For each class *k*, the most central element *ω*^*k*^ is chosen to define the representative set, hence: Ω_*K*_ = {*ω*^1^, …, *ω*^*K*^}.

### 3.2 Non-parametric reconstruction of distributions

Now, we assume that when a new input **x** is considered, one may compute the outputs corresponding to the representative set: *y*(**x**, Ω_*K*_). The next step is to obtain accurate estimations of the objective functions E[Y(x)] and CVaR_*α*_[*Y*(**x**)] based on these values.

**Fig 3:**
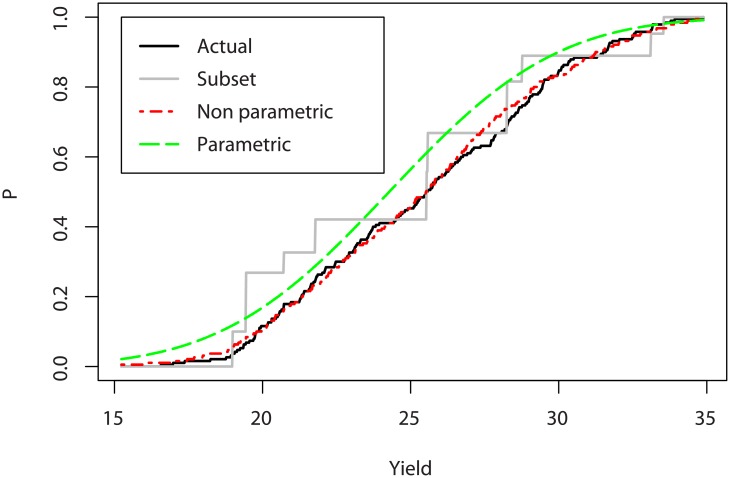
Actual and estimated CDFs of the yield of a given phenotype.

Computing directly the objective functions would lead to large errors, in particular for CVaR_*α*_[*Y*(**x**)], that requires an accurate representation of the tail distribution. A natural alternative is to infer a distribution on the small data set, and compute the objectives on the distribution. However, in the sunflower case the form of the empirical distribution (Fig 3) does not readily call for a given parametric model, and misspecifying the distribution shape may result with large bias.

Hence, we propose to infer the distribution using a non-parametric method, by re-using the data computed for the classification step, that is, the output matrix y(B,Ω). To do so, we assume a mixture model for *y* (each component corresponding to a class $C^{k}$):

$$f_{Y(x)}\left(y\right)=\sum_{k=1}^{K}\frac{N^{k}}{N}f_{Y^{k}\left(x\right)}\left(y\right),\;y\in R,$$ (7)

*f* standing for the probability density function and *Y*^*k*^(**x**) being the output within the class *k*.

We decompose further *Y*^*k*^(**x**) as the sum of the output value at the representative element and a residual term:

$$Y^{k}\left(x\right)=y\left(x,\omega^{k}\right)+\varepsilon^{k}\left(x\right).$$ (8)

The intra-class distribution is then characterized by the residuals *ε*^*k*^(**x**), which determine the form, spread (or amplitude), and bias (i.e., difference between the average value and the value for the representative element). All these elements vary from one class to another, which advocates the use of non-parametric approaches. To model *ε*^*k*^(**x**), we introduce the weighted variance of the yield over the representative set:

$$\begin{array}{c} \sigma_{K}^{2}\left(x\right)=\frac{1}{N}\sum_{k=1}^{K}N^{k}{\left(y\left(x,\omega_{k}\right)-\frac{1}{N}\sum_{j=1}^{K}N^{j}y\left(x,\omega_{j}\right)\right)}^{2}. \end{array}$$ (9)

Note that for a new phenotype **x**, the only data available is *y*(**x**, *ω*_*j*_). We then define averages of *normalized* residuals:

$${\bar{\varepsilon }}^{k}=\left[{\bar{\varepsilon }}_{1}^{k},\ldots ,{\bar{\varepsilon }}_{N^{k}}^{k}\right]\text{,}\;\text{with}\;{\bar{\varepsilon }}_{j}^{k}=\frac{1}{l}\sum_{i=1}^{l}\frac{\varepsilon_{j}^{k}\left(x_{i}\right)}{\sigma_{K}\left(x_{i}\right)}.$$ (10)

and the output is approximated, with *i* uniformly taken from ⟦1, *N*^*k*^⟧, by:

$${\hat{Y}}^{k}\left(x\right)=y\left(x,\omega^{k}\right)+\sigma_{K}\left(x\right)\times {\bar{\varepsilon }}_{i}^{k}.$$ (11)

**Fig 4:**
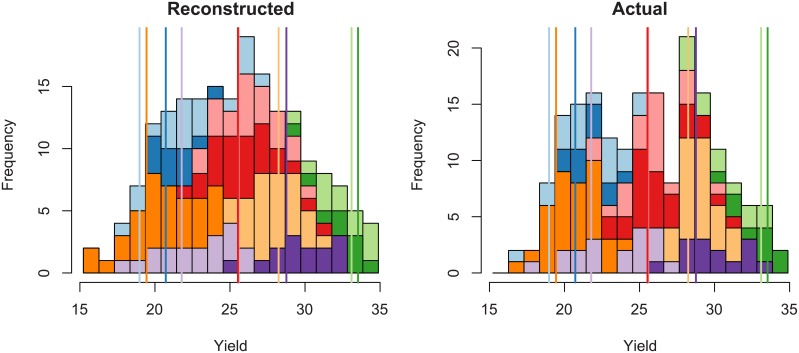
Estimated yield distribution of a given phenotype. The colours show the different classes, and the vertical bars the output values for the representative elements.

Fig 4 illustrates the reconstruction technique for a given (randomly chosen) phenotype, by showing how the residuals corresponding to each class are used to obtain the estimated distribution. We can see that the range and shape of the residuals vary considerably from one class to another. Also, their distributions around the representative element differ: as the residuals do not have a zero mean, the value of the representative element is not necessarily central for each class. Comparing the reconstructed (Fig 4, left) and actual (right) distributions, we see that the mixture is globally the same on both graphs.

Fig 3 shows the cumulative distribution function (CDF) of the actual yield and of two estimations: using the method described above and a simple parametric method, which consists in assuming a Gaussian distribution of the yield. The empirical CDF corresponding to the subset values only is also depicted, with unequal steps to account for the different number of elements in each class.

We first notice that the subset data is by itself insufficient to evaluate accurately the mean or the CVaR. Then, we see that the actual distribution does not seem to belong to a known distribution, and using a normal distribution introduces a large bias. Inversely, using a non-parametric reconstruction allows us to match the shape of the actual distribution.

In our study, we found that this reconstruction method provided a satisfying trade-off between robustness, simplicity and accuracy. Yet, many refinements would be possible at this point, for instance by introducing intra-class rescaling (different normalization for each class), bias correction, or using the distance from the phenotype **x** to the basis B.

### 3.3 Optimization: A two-step approach

Finally, the multi-objective optimization problem can be solved with any black-box algorithm for $E[\hat{Y}\left(x\right)]$ and ${\text{CVaR}}_{\alpha }[\hat{Y}\left(x\right)]$, with $\hat{Y}\left(x\right)$ a mixture of ${\hat{Y}}^{1}\left(x\right),\ldots ,{\hat{Y}}^{K}\left(x\right)$. However, the objective estimates are based on the phenotype basis B, which is sampled uniformly over X to offer a general representation of the phenotype space. This feature is important at the beginning of the optimization to ensure that the optimizer does not get trapped into poorly represented regions. However, as the optimizer converges towards the solution, the search space becomes more narrow, and a substantial gain in performance can be achieved by modifying the estimates so that they are more accurate in the optimal region.

The strategy we adopt to overcome this problem is to introduce, during the optimization procedure, a step of re-evaluation of averages of normalized residuals ${\bar{\varepsilon }}^{k}$ (Eq 8). First, we run the optimization with the initial basis B. Then, we select *l* new phenotypes from the obtained Pareto set to form $B^{\prime }$ and we evaluate $y(B^{\prime },\Omega )$ and the new version of ${\bar{\varepsilon }}^{k}$ by performing *N* × *l* simulations. Finally, we restart the optimization with the new estimates of ${\bar{\varepsilon }}^{k}$. We have found (Section 5) that this two-step strategy was sufficient on our problem, while relatively easy to implement.

## 4 Experimental setup

### 4.1 Climatic dataset

**Fig 5:**
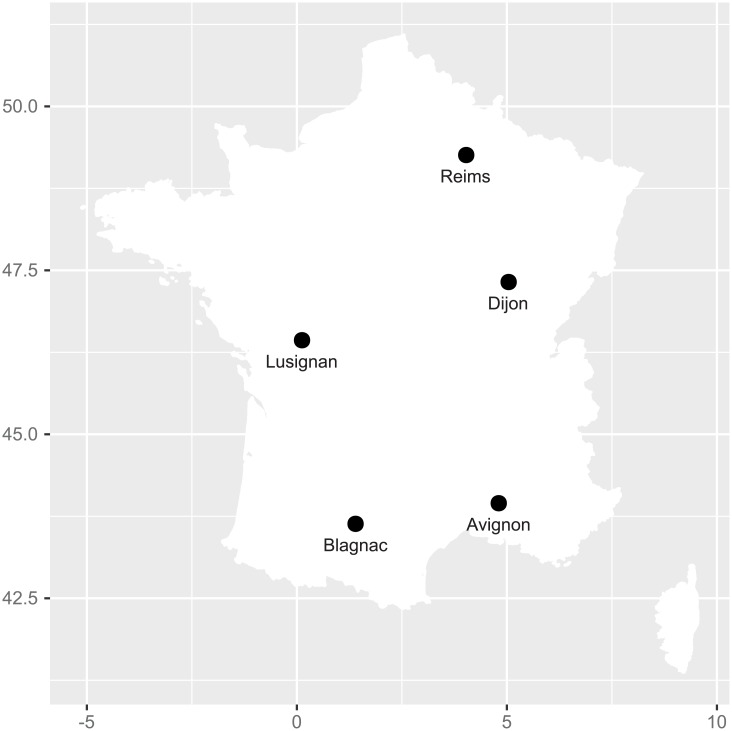
Location of the five French stations for the historic climatic data.

We used historic climatic data from five French locations where sunflower crop frequent (Reims, Dijon and Lusignan that are in the north of the country, and in Avignon and Blagnac in the south, see Fig 5) from 1975 to 2012. This resulted in a set Ω of size *N* = 190.

### 4.2 Climate subset selection

We used the R package dtw [26] to compute the distances. The window size (that is, the maximum shift allowed) is the main influential parameter of the method. We used here expert knowledge to choose it based on the sunflower phenology: for precipitation, a window of ±3 days is used; for the other variables, a window of ±7 days is chosen (e.g. we considered that a weekly shift on temperature changes little the yield). The phenotype set B is chosen as a 10-point LHS; hence, for this step the method required 1,900 calls to the SUNFLO model.

Then, a k-means algorithm is run. Since it provides a local optimum only, we performed several restarts to achieve a good robustness. Several number of classes *K* were tested; we found empirically that *K* = 10 provided a satisfying trade-off between the representation capability of the subset and the computational cost during the optimization loop.

### 4.3 Optimization setup and comparison benchmark

To assess the validity of our approach, we conducted an empirical comparison with two alternatives: a random search and a black-box optimization, both based on the full set of climate series. We compare the different approaches based on an equal number of calls to SUNFLO (that is, we do not consider the other time costs related to each approach). We consider three budgets, which we refer to as large (380,000), medium (95,000) and small (23,750).

Our approach (denoted *two-step* in the following) uses the MOPSO-CD metaheuristic (Multi-Objective Particle Swarm Optimization with Crowding Distance, [27]), available in the R package mopsocd. Its two main parameters are the population size and number of generations (their product being equal to the number of function evaluations, i.e. the *budget*). A standard optimization based on all the series is also performed using the original MOPSO-CD algorithm (henceforth *full MOPSO*). Random search is based on optimized Latin hypercube sampling (LHS) to fill the search space X using the R package lhs.

For each budget, we define the number of iterations and the population size for the full and two-step approaches. As a rule-of-thumb, we set the number of iterations to approximately five times the population size [28]. For the two-step algorithm, each evaluation of the objectives requires ten SUNFLO runs (while the other approaches require 190 runs), which allows a larger population and number of iterations, but the initial and intermediate learning steps have a 10 × 190 cost. Table 2 reports the detailed setups for each budget and algorithm.

**Table 2:** Parameters of the different approaches algorithms depending on the computational budget.

Optimization experiment	Budget	Nb of iterations	Pop size	Real nb of simulations
**Random (or LHS)**	small	-	125	23,750
	medium	-	500	95,000
	large	-	2,000	380,000
**Full MOPSO-CD**	small	25	5	24,700
	medium	50	10	96,900
	large	100	20	383,000
**Two-step MOPSO-CD**	small	71(×2)	14	23,960
	medium	152(×2)	30	95,600
	large	308(×2)	61	380,780

Since all the algorithms used are stochastic, each optimization experiment is replicated ten times to assess the robustness of the results.

## 5 Results and discussion

### 5.1 Climate subset selection

**Fig 6:**
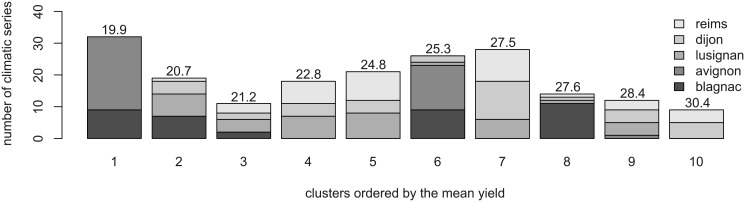
Clusters split by locations in France. Average yields, computed on the base B and the set of climates, are given above the bars.

Fig 6 shows the clusters obtained with the approach described in Section 3.1. in terms of location and average yield. Three clusters (1, 6 and 8) consist of a majority of series from the south of France (Avignon and Blagnac, hence on average hotter and drier), four from the north (4, 5, 7 and 10) and three with mixed locations.

The average yield provide a complementary information on the clusters. Overall, the clusters cover a large range of yields, which distinguishes series from the same locations (1 and 5 or 4 and 9). Note that integrated quantities, such as evapotranspiration annual average do not explain well the different classes. Especially, there is a known high impact of rain episodes and their location in time, which may be “seen” by our composite distance, but is challenging to display here.

### 5.2 Phenotype optimization

#### 5.2.1 Algorithm performance assessment

**Fig 7:**
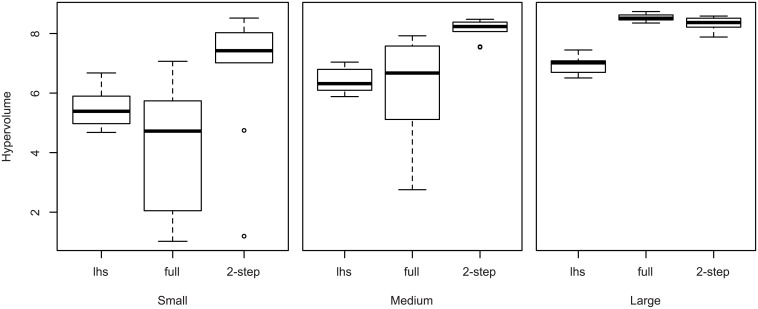
Performance of the different methods for the three budgets considered.

Now, we compare the performances of the approaches described in Table 2. We use a classical multi-criteria indicator (hypervolume) that measures the volume of the objective space behind the Pareto front (the larger the better, see for instance [29] for details). Fig 7 show the indicators of the different runs in the form of boxplots.

For the small and medium budgets, the two-step approach clearly outperforms the other approaches (with the exception of two outliers with the small budget). For the large budget, we see that the regular MOPSO-CD performs slightly better, which is expected. Indeed, as soon as there is no necessity of parsimony, using approximate objectives instead of actual ones tends to slow down, rather than accelerate, convergence. However, we can conclude that our two-step approach with medium budget performs almost as well as the regular approach with large budget, hence for a quarter of the budget. In the small budget case, which is likely to happen when more expensive environmental models (time-wise) are considered, our approach still provides reasonably good results, while the classical optimization fails at providing a better performance than random search.

#### 5.2.2 Sunflower ideotype analysis

**Fig 8:**
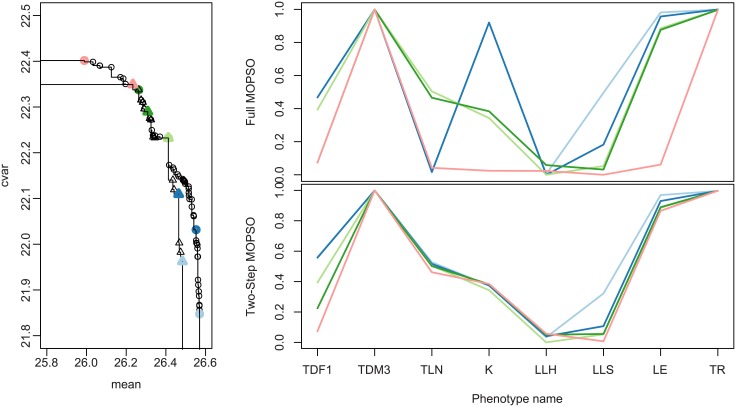
Left: Pareto fronts obtained by the full MOPSO-CD (circles) and the two-step (triangles) approaches, with large and medium budgets, respectively. The bold symbols correspond to a subset of five optimal phenotypes that are shown on the figures on the right, where each line represents a phenotype.

Finally, we characterize the results on the phenotype space. We compare here the Pareto set obtained by merging all the runs (which can be considered as close to the actual solution) with one run of the two-step method; we chose the run on the medium budget with the median performance. For readability, we only consider a subset of the Pareto set of size five, equally spaced along the Pareto front. The Pareto fronts and sets are represented in Fig 8.

We can see first that considering both the expectation and CVaR for optimization leads to a large variety of optimal phenotypes. Looking back at the plant characteristics corresponding to those solutions, the optimum value for three traits had little variability, meaning that those traits were important plant characteristics for crop performance in the tested environments. Those traits depicted plants adapted to water deficit: a late maturity (TDM3), largest leaves at the bottom of the plant (LLH) and a conservative strategy for stomatal conductance regulation (TR). The five other traits (TDF1, TLN, K, LLS, LE) displayed variability in optimal values, which was identified as the basis of the performance/stability trade-off (expectation/CVaR). Here, the traits (except TLN) vary monotonically along the Pareto front.

Distinct plant types could be identified in the phenotype space. For example, the *pink-red* plant type had an early flowering (TDF1), a low light extinction efficiency (K) and a low plant leaf area (LLS); those characteristics correspond to a conservative resource management strategy. In an opposite manner, the *light-blue* type displays a late flowering, a high efficiency to intercept light and a larger plant leaf area, characteristics usually associated with a productive but risky crop type when facing strong water deficit [30]. The strategy associated with plant types identified from the phenotype space matched their position in the Pareto front, i.e the *light-blue* plant type was more efficient but less stable than the *pink-red* one.

The Pareto set obtained with the two-step method reproduces part of these features: the fixed traits are similar (except TLN, which is fixed to approximately 0.5, but this parameter is known to have little impact on the yield, see [16]) and the variation of TDF1 and LLS is well-captured. However, on this run the method failed at finding the variation of the K and LE traits: this probably explains why the largest mean and CVaR values (extremities of the actual Pareto front) are missed.

Overall, the two-step method allowed to identify the few key traits that are responsible for the cultivar global adaptation capacity as well as secondary traits that support alternative resource use strategies underlying the yield expectation/stability trade-off.

## 6 Summary and perspectives

In this article, we proposed an algorithm for the optimization of black-box models with uncertain climatic inputs, and applied it to the design of robust sunflower cultivars. Our approach does not require any *a priori* knowledge on the system besides parameter bounds, hence is usable with most simulators depending on similar climatic data. Using subset selection for the climates allowed us to reduce substantially the computational time without adding implementation issues. If bias correction seems inevitable during optimization, we showed that a two-step strategy was sufficient to achieve convergence: this point is critical as it allows our approach to be combined with any black-box multi-objective solver.

Nevertheless, we see many opportunities for further improvements. First, one could study the impact of the number of clusters on the results, which is a recurrent question with clustering methods. Second, a popular strategy to reduce the computational costs is to combine optimization with the use of surrogate modelling (see for instance [1]). Our approach straightforwardly extends to such approaches, and would result in very parsimonious algorithms that may be beneficial for expensive simulations. Finally, we have chosen here to use a two-step strategy to allow the use of “off-the-shelf” optimization solvers. Interlinking optimization and learning may improve substantially the efficiency of the method, although requiring the development of an *ad hoc* algorithm.

## Supporting information

S1 Dataset and Code Data and software instructions for implementing computer experiments. The dataset used throughout this manuscript and the R code used to call the SUNFLO model and run the proposed algorithm are provided in a single archive file.
(TGZ)
